# Spatiotemporal elements in a poisoned bait strategy against the long-tailed silverfish (Lepismatidae: Zygentoma)

**DOI:** 10.1371/journal.pone.0260536

**Published:** 2021-11-29

**Authors:** Bjørn Arne Rukke, Morten Hage, Anders Aak

**Affiliations:** Department of Pest Control, Norwegian Institute of Public Health, Oslo, Norway; King Khalid University, SAUDI ARABIA

## Abstract

The long-tailed silverfish *Ctenolepisma longicaudatum* (Lepismatidae: Zygentoma) is a nuisance problem in buildings and a major concern in museums, libraries and archives where it cause damage to historical and priceless items. We used laboratory bioassays and two field studies of infested buildings to evaluate spatial and temporal elements of a poisoned bait strategy. In both laboratory experiments and field studies, the efficiency of poisoned bait with indoxacarb as the active ingredient was significantly improved by placing many small bait droplets evenly distributed along all edges of the treated area compared to more clustered distributions. Extended duration of bait presence and removal of competing food sources improved the control effect significantly in the laboratory bioassays. Bait-treated populations also showed a significant decline in the number of eggs deposited and emergence of new nymphs. The study supports poisoned bait as an efficient and low risk approach against the long-tailed silverfish in which extended duration of bait presence, wide distribution of bait droplets in combination with sanitation was crucial for control in the infested premises.

## Introduction

The long-tailed silverfish (*Ctenolepisma longicaudatum* Escherich, 1905), formerly known as *longicaudata* [1,2], Zygentoma; Lepismatidae) has recently emerged as a nuisance indoor pest in several European countries [3–16]. They cause minimal physical damage in private homes, but due to the psychological discomfort from them crawling around on floors, walls, furniture and other objects, they are considered a nuisance problem, particularly in new and otherwise immaculate buildings [8,15]. The *C*. *longicaudatum* is known to easily spread throughout buildings due to the small size of mobile nymphs, active adult dispersal or hitchhiking with objects [15,17]. The species feeds on a variety of items like paper, pictures, old books, plant-based cloth and protein-rich sources such as dried meat or dead insects [13,18–20]. In museums, libraries and archives it is of major concern because of potential damage by foraging on historical and priceless items [21–24].

There is a need for the development of efficient and safe control methods *for C*. *longicaudatum*, and concerns regarding indoor use of pesticides point at the need for caution and moderation in choice of strategy. *C*. *longicaudatum* often has a building-wide distribution [13,17], and conventional spray pesticides require applications throughout the infested premises. In such situations, control by bait is preferable to sprayable pesticides because it limits the potential negative impact of pesticide exposure [25,26]. Recent laboratory and field experiments have identified commercially available insecticidal baits (hereafter denoted as baits only) as effective against *C*. *longicaudatum* both through direct consumption and by secondary poisoning when dead conspecifics are consumed [17,27,28].

Behaviour, food preference and spatial distribution of indoor pests are important factors to consider when choosing a control strategy. *C*. *longicaudatum* is nocturnal [29,30], use cracks and crevices for movement or hiding and assemble in loosely defined and partially hidden aggregations [19]. Once established in a building, *C*. *longicaudatum* appears to have a more uniform distribution in both dry and humid rooms [17], as opposed to the common silverfish (*Lepisma saccharinum*) or the firebrat (*Thermobia domestica*) that typically appear in excessively humid and hot environments, respectively [13,31]. The biology of *C*. *longicaudatum* partially overlaps with two commonly encountered indoor pests, cockroaches and pharaoh ants, that both are efficiently controlled by baits [18,19]. Pharaoh ants may dynamically distribute their sub-populations into all suitable locations in a building [19,32] whereas control of cockroaches may have a more local character when they form breeding and feeding aggregations close to foods sources [32]. From a bait control perspective, *C*. *longicaudatum* appears to resemble pharaoh ants in their ability to disperse widely in the building and establish at many different locations, whereas their foraging appears closer to cockroaches because individuals tend to proliferate around permanently present food sources [18,19].

*C*. *longicaudatum* appears to experience little spatial restrictions and shows a uniform distribution in buildings [17]. This might be a challenge for control with baits because reaching all individuals in the population can be difficult. However, efficiency of a bait strategy is also likely to be affected by removal or changed availability of food sources [33–38]. Successful bait application may therefore be achieved by use of a building-wide approach and strategic bait application similar to pharaoh ant control, in combination with a cockroach control-like sanitation strategy, to remove alternative and competing food sources [17].

As a part of an initiative to develop an efficient and safe control strategy against *C*. *longicaudatum* in Norway [13,15,17,27,39], we evaluated spatial and temporal variations in bait placement in combination with sanitation. First, the study uses laboratory bioassays to evaluate the spatio-temporal elements on both adult and juvenile survival. This is done by varying either the positioning of bait droplets in large arenas simulating a room or by altering the duration of bait presence in smaller experimental arenas simulating a foraging situation. Secondly, we describe two full-scale case studies where control of infested buildings is achieved.

## Materials and methods

### Stock cultures

Stock cultures of *C*. *longicaudatum* were initially established by collecting more than 40 individuals from each of four localities in and around Oslo city, Norway, in 2016 and 2017. Cultures were maintained and allowed to breed freely in plastic terraria (mouse cage bottoms; Innovive, San Diego, CA, USA) with a sheet of paper on the floor, various available hiding places and a continuous food supply of goldfish flakes (Astra Goldfish flake; Astra Aquaria GmbH, Melle, Germany) and flaked oat (Axa, Oslo, Norway). The stock cultures had constant access to moisture through water-filled glass tubes closed with a cotton wick, and dry cotton was provided as an egg deposition substrate. The rearing room with all stock cultures was maintained at optimum conditions for *C*. *longicaudatum* (24–26°C, 60% relative humidity and 16:8 h (light:dark) cycle). As the stock populations increased in density, the cultures were divided into multiple cultures from which we could collect freshly moulted individuals for experiments.

### Poisoned bait

Advion® Cockroach Gel (0.6% indoxacarb; Syngenta, Basel, Switzerland) was chosen as the test bait, as it is approved for silverfish control in EU and EEA [40] and has been demonstrated to be effective against *C*. *longicaudatum* in laboratory and field situations [17,27].

### Bait placement–spatial elements

**Fig 1:**
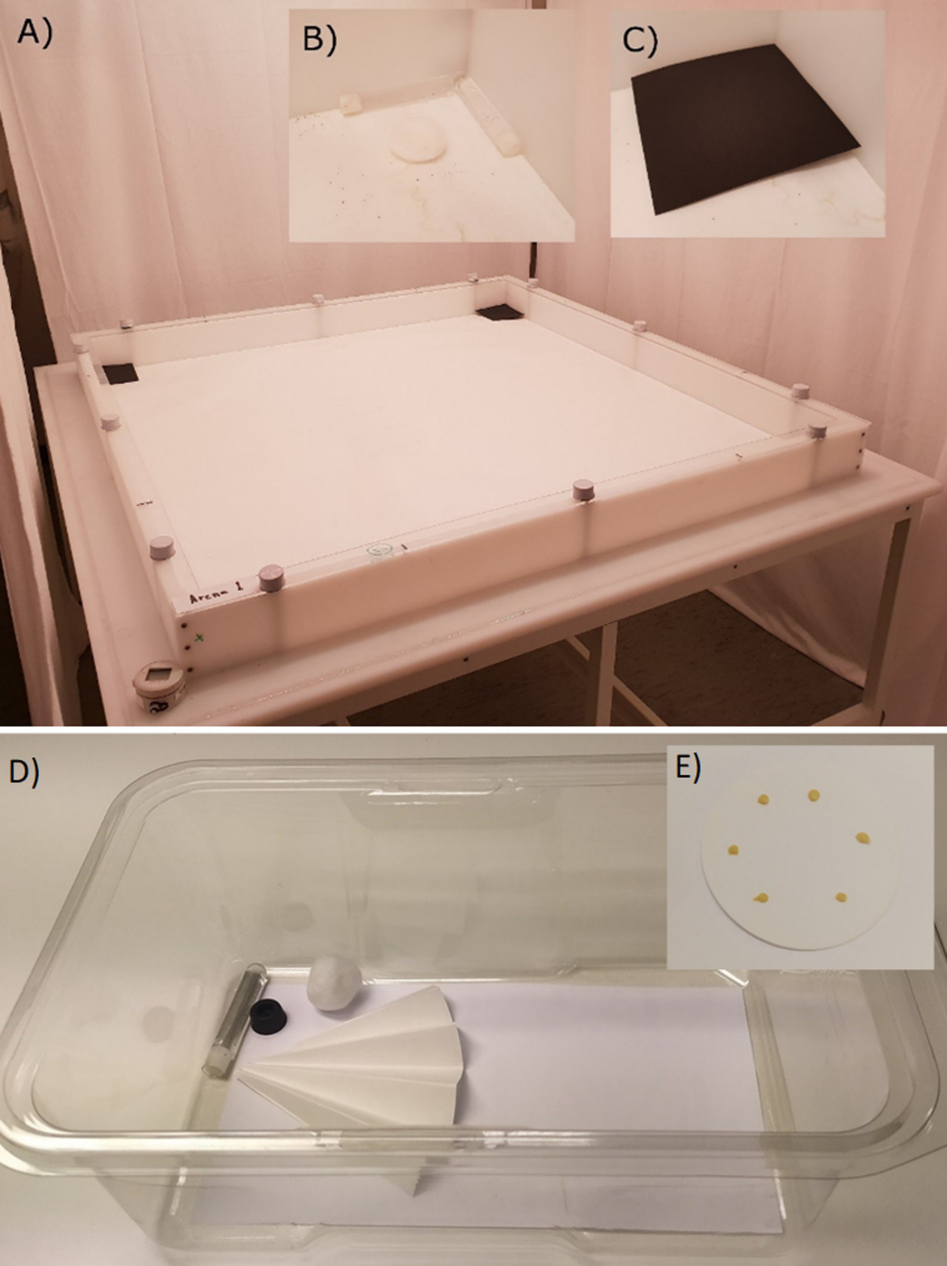
Bioassays used to test bait against *Ctenolepisma longicaudatum*. (A) An overview of the arena with an inner experimental area of 126 cm × 126 cm used to test spatial elements of bait placement, (B) water tubes and cotton ball in harbourages and (C) harbourage with black lid in place. (D) Population boxes used to investigate temporal elements of bait placement and sanitation. (E) Filter paper with six droplets of Advion® Cockroach Gel bait that were placed in the population boxes for test of temporal elements of bait placement.

Effects of spatial variation of bait placement were explored in arenas originally designed for behavioural studies of bed bugs. The arena represent a simulation of a room (126 × 126 cm, Fig 1A) by allowing insects to move freely between their hiding places and open spaces. The details of construction and hardware are thoroughly described in previous publications [41,42]. However, the harbourages had to be modified and made more suitable for *C*. *longicaudatum*. This was done by including two water-filled glass tubes closed with cotton wicks and a dry cotton ball for egg deposition (Fig 1B) in each harbourage. On top of the tubes and the cotton ball, we placed a 11 cm × 11 cm sheet of black cardboard to create a harbourage with darkness and access to moisture. As a result of the placement of tubes and the cotton ball, the cardboard was tilted slightly to let the corner pointing towards the centre of the arena touch the paper floor (Fig 1C). This also created a variation in height of the harbourage roof reaching from 0 to approximately 10 mm. The arena room maintained a 16:8 h light:dark cycle, a temperature of 24.0 ± 0.0°C and 53.7 ± 0.3% relative humidity to match our rearing conditions and the preferred conditions for *C*. *longicaudatum* [30].

We used five pairs of *C*. *longicaudatum* per arena. Because transition from juvenile to adult progress gradually between stage 8 and 14, and moulting continue after maturation, adulthood was determined by body size > 11 mm, and gender according to presence/absence of ovipositor. The 10 adults were gently collected from the stock cultures by letting them crawl onto a piece of paper and transferring them to a new terrarium with only a paper floor, a water tube and a harbourage made from ¼ of a pre-folded filter paper (Whatman 2555 ½; GE Healthcare, Buckinghamshire, UK). The adults were kept in this terrarium for 4 days (to ease logistics and allow time-effective use of the large arenas) before being transferred to the arena where they were given 3 days to establish aggregations in the harbourages and acclimatize. Bait was then placed in the arena according to the experimental design. At the time of bait introduction, the adults had been starved for 7 days and were then given 4 days to find and consume the bait before mortality was registered. No competing food source was present in the arena.

Four experimental treatments with different placement of 12 droplets of bait were investigated (average droplet weight = 5.4 ± 0.2 mg). Treatment 1 had all 12 droplets placed close together (1 cm apart) in the centre of the arena. Treatment 2 had the 12 droplets evenly distributed in the whole arena (within the 80 cm × 80 cm centre square). Treatment 3 had three droplets placed close together (1 cm apart) at the centre of each of the edges of the arena. Treatment 4 had three droplets evenly distributed (40 cm apart) along each of the four edges of the arena. The four experimental treatments were replicated six times to supply a total of 240 individual *C*. *longicaudatum* across the entire experiment and 60 individuals per treatment.

### Bait placement–temporal elements

Effects of time with access to bait were investigated in small plastic terraria (mouse cage bottoms) with a 14.5 cm × 27 cm premium label paper (Herma GmbH, Filderstadt, Germany) adhered to the floor (Fig 1D). In one end of these population boxes, we placed a glass-tube water station, a dry cotton ball (0.5 g; Cutisoft, Hamburg, Germany) for egg deposition and one-quarter of a pre-folded filter paper, diameter 240 mm (Whatman 2555 ½) as harbourage. The opposite half of the population boxes was used to present the food and bait according to the experimental design.

As a representation of a natural population, we selected 24 *C*. *longicaudatum* individuals from the stock cultures. The sex ratio was balanced with six male and six female adults. Adults were supplemented with six large juveniles with a body length within 7–11 mm and six juveniles smaller than 5 mm. The adults and juveniles were gently picked up by letting them crawl onto a piece of paper before transferring them to their experimental terraria. They were allowed to establish in the terraria for 14 days before introducing the bait. Throughout the entire experiment the population boxes had surplus amounts of goldfish flake and ecologic flaked oat to supply required nutrients and as a competing food source for the bait. To replicate droplet size of an ongoing large scale field experiment [17], bait was introduced into each box as six droplets on a filter paper (average droplet weight = 7.99 ± 0.46 mg, Fig 1E) placed among the competing food sources. This allowed removal of the bait after the pre-determined time in the box. During the experiment, mortality was registered bi-weekly on Mondays and Thursdays until day 63 and then weekly on Mondays until termination of the experiment on day 112. Dead specimens were not removed to allow for secondary poisoning. During registration of mortality, we also counted the number of nymphs hatched from deposited eggs and recorded their developmental stage. On termination of the experiment, the total number of eggs deposited were counted.

Four initial experimental treatments investigated the effect of the duration of bait presence. Bait was placed in the terraria and removed after either 2, 8, 16 or 32 days. One additional 8-day treatment, in which bait was discontinuously present in four 2-day periods, 6 days apart, was compared to the continuous 8-day treatment. To simulate the impact of a thorough vacuum cleaning regimen prior to bait application, we used a second 2-day treatment. In this treatment, food was removed 4 days prior to introduction of the bait. All six experimental treatments used six population replicates (population boxes) each for comparison with six control population boxes without bait. The total number of specimens at the start of the experiment was 1008 with 144 specimens in each treatment.

### Case studies–field observations on spatial elements

To develop and evaluate field strategies for control, two buildings with *C*. *longicaudatum* infestations were treated with bait. This work was done alongside the laboratory experiments, and the field trials were adjusted according to the laboratory findings when results were considered crucial for efficient control.

**Fig 2:**
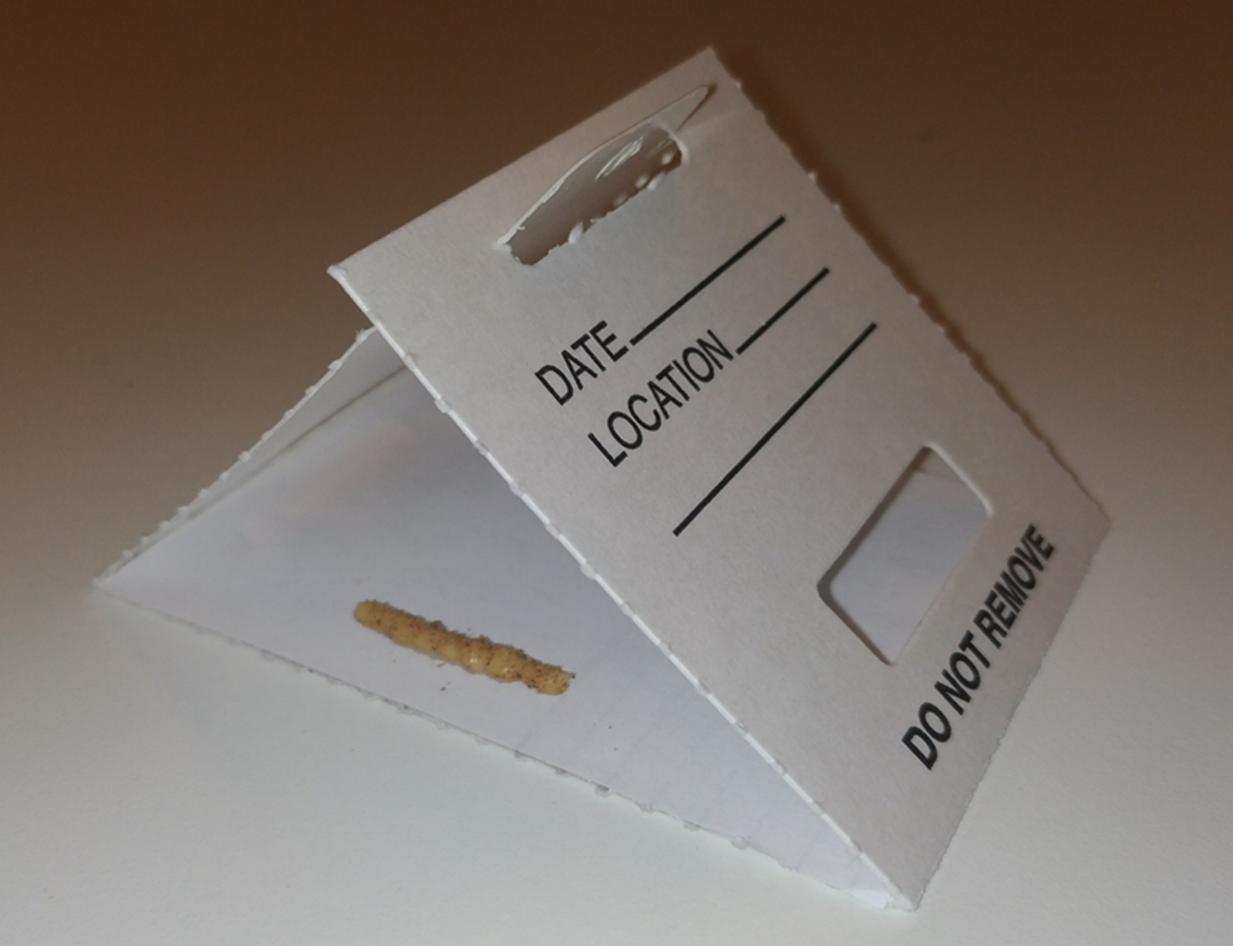
Bait station for *Ctenolepisma longicaudatum* made from a deactivated delta trap. Bait is sprinkled with cricket powder to act as an attractant.

The first infested site was a large three-storey detached house treated with open-sided bait stations (Fig 2). Each room in this building held 1–3 bait stations. This can be considered a representation of the two centered treatments in our laboratory arena study with only 1–4 points of bait access. Bait was replenished every 4^th^ week to ensure freshness, and we used small amounts of cricket powder (100% Acheta cricket, Unik mat, Asker, Norway) sprinkled on the surface of the bait to act as an attractant, meant to increase consumption of the bait [13]. The effect on the *C*. *longicaudatum* population was measured by 94 sticky traps (trapper monitor and insect glue traps; Killgerm, Ossett, UK) evenly distributed throughout the building because these traps are known to have a limited and non-significant impact on population density in well-established populations [17]. Traps were in place for 14 days prior to bait application to obtain a pre-treatment measurement and then replaced every second week for the first year. Thereafter, traps were present for 2 weeks in every two months until no *C*. *longicaudatum* was found. After an initial 50 weeks with limited effect from the bait station approach (1–3 stations per room) and after obtaining knowledge regarding spatial effects through the laboratory experiment, we switched to a strategy with many small droplets distributed along the wall skirtings throughout the building to mimic the evenly distributed edge treatment in our laboratory study. All bait stations were removed at this point and approximately 100 small droplets per 100 m^2^ were placed throughout the building.

The second infested site was an apartment complex with known established populations of *C*. *longicaudatum* in 49 out of 57 (86%) apartments. Apartment size ranged from 50–110 m^2^. By consideration of the laboratory effects and a possible improved efficacy observed in the single house, the entire building was treated using many small droplets placed along the wall skirtings throughout the building at approximately 100 small droplets of bait per 100 m^2^. Bait without any additional attractant was mainly placed in cracks and crevices along walls and underneath fixed furniture and appliances. The effect on the *C*. *longicaudatum* population was monitored closely in nine infested apartments with a total of 120 traps evenly distributed between the apartments. Traps were in place for 14 days prior to bait application and then for 14 days after 1, 10, 20 and 28 weeks.

### Statistical methods and analyses

Data were analysed using SigmaPlot 14.0 (Systat Software Inc. San Jose, CA, USA) and JMP pro 15.0.0 (SAS Institute, Cary, NC, USA) software [43,44]. The level of significance was set to 0.05, and averages were presented with standard errors (±SE). Multiple comparisons were performed using one- or two-way analysis of variance (ANOVA) followed by the Holm–Sidak method or Tukey test for multiple comparisons. For the initial temporal experiments, offspring production was analysed by grouping the 112 experimental days into five periods of 3 weeks, and the number of living 1^st^ to 3^rd^ instar nymphs in each of the six population boxes was used as the response variable. The Kaplan–Meier product limit method with the log-rank test between groups was used for survival analyses. To adjust for multiple pairwise comparisons of experimental treatments in survival analyses, Bonferroni corrections were performed to correct the level of significance.

## Results

### Bait placement–spatial elements

**Fig 3:**
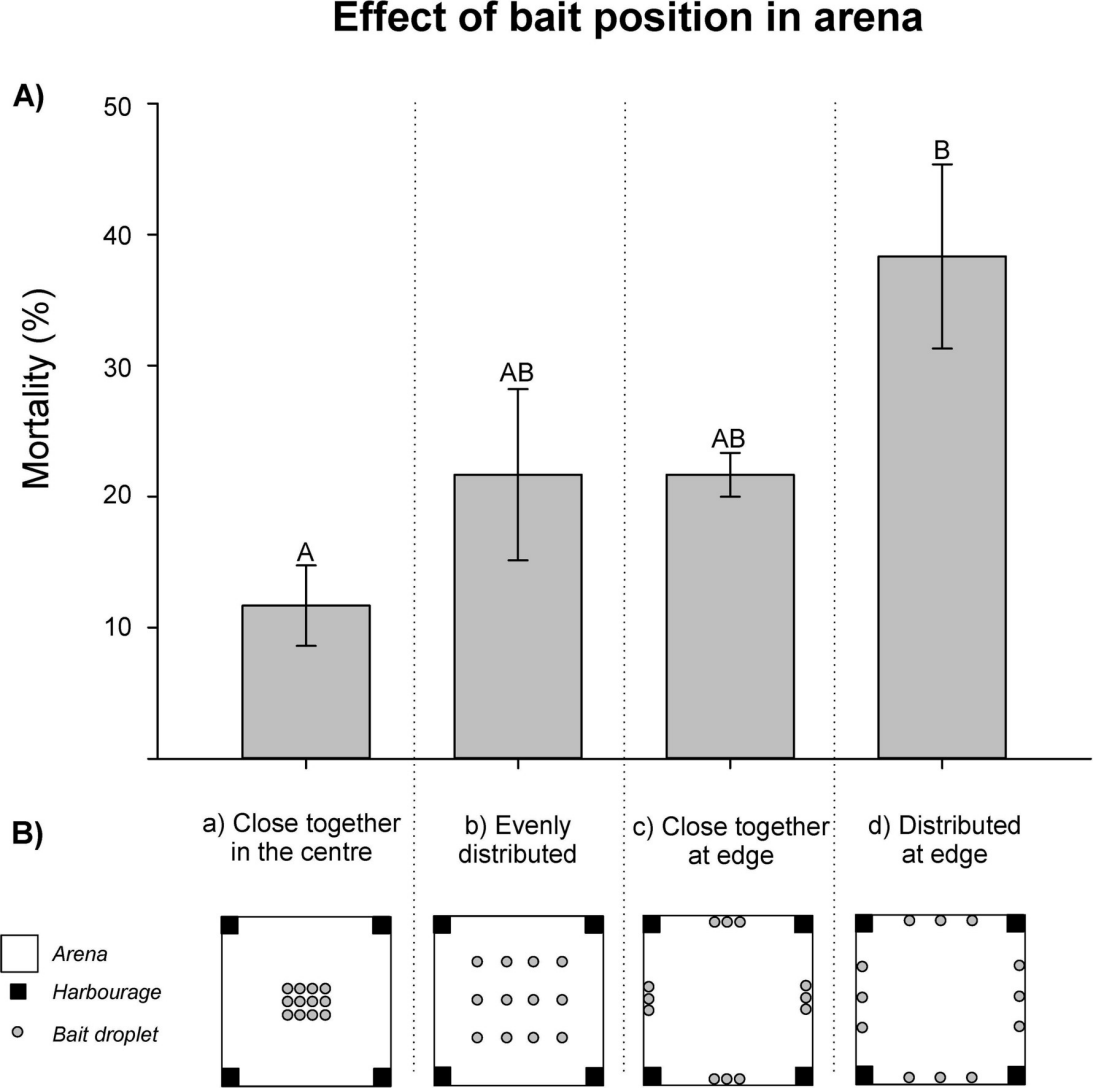
A) Mortality of *Ctenolepisma longicaudatum* in arenas for four experimental treatments with different spatial distribution of 12 poisoned bait droplets. Different letters (A and B) indicate significant differences in mortality between pairs of treatments (Tukey test, p < 0.05). B) 12 droplets of bait placed a) 1 cm apart in the centre of the arena, b) evenly distributed within an 80 cm × 80 cm square in the middle of the area, c) as three droplets (1 cm apart) at the four perimeter edges or d) as three droplets evenly distributed (40 cm apart) at each perimeter edge.

The efficiency of baits was significantly improved by approximately four times when we distributed the bait droplets alongside the edges of the arena compared to placement close together in the centre (ANOVA: *F* = 4.68, p = 0.012) and mortality was twice as high as the intermediately effective strategies with evenly distributed droplets and droplets close together at the edges (Fig 3).

### Bait placement–temporal elements

**Fig 4:**
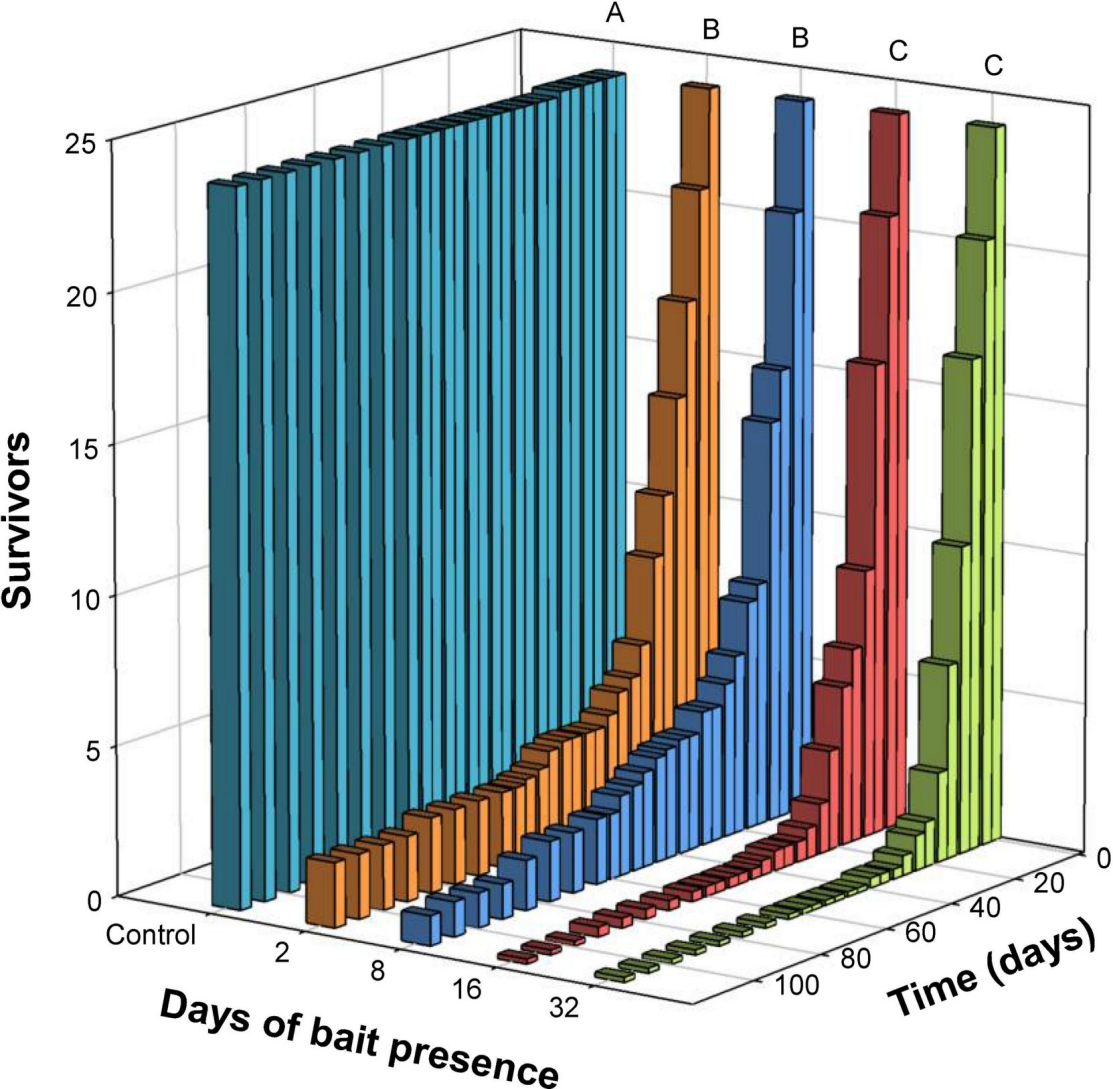
Different bait placement duration and survival of *Ctenolepisma longicaudatum* in population boxes. Different letters (A, B and C) indicate significant differences in survival between pairs of treatments (Kaplan–Meier survival analyses, p < 0.05).

All *C*. *longicaudatum* life stages were pooled for the analyses because there were no differences in survival between small nymphs, large nymphs and adults across the six bait treatments (Kaplan–Meier; χ^2^ = 0.24, df = 1, p = 0.888). Treatments with bait continuously present for 2, 8, 16 or 32 days reduced survival significantly compared to the control (Kaplan–Meier; only least significant test shown, χ^2^ = 268.5, df = 1, p < 0.001, Fig 4). Bait presence of 2 and 8 days did not differ from each other (Kaplan–Meier; χ^2^ = 0.30, df = 1, p = 0.303), but these two short-term treatments experienced significantly higher survival compared to 16 and 32 days of bait presence (Kaplan–Meier; only least significant test shown, χ^2^ = 9.64, df = 1, p = 0.002). Bait presence of 16 and 32 days yielded comparable survival (Kaplan–Meier; χ^2^ = 0.57, df = 1, p = 0.450).

**Fig 5:**
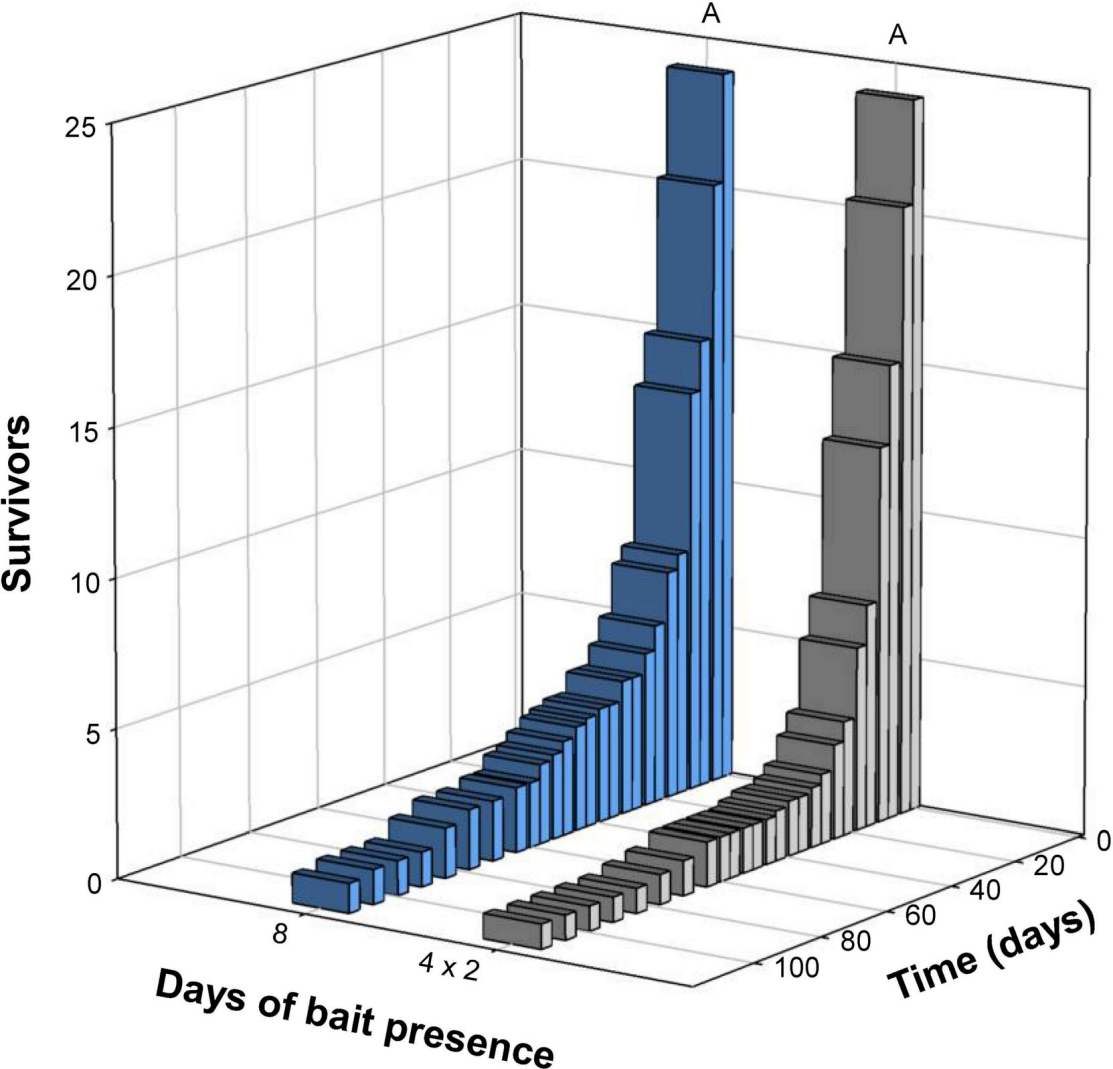
Survival of *Ctenolepisma longicaudatum* in population boxes of the 8-day continuous bait treatment and the 4 × 2-day bait treatment, respectively. The same letter (A) indicates a non-significant difference in survival between the two treatments (Kaplan–Meier survival analyses, p > 0.05).

**Fig 6:**
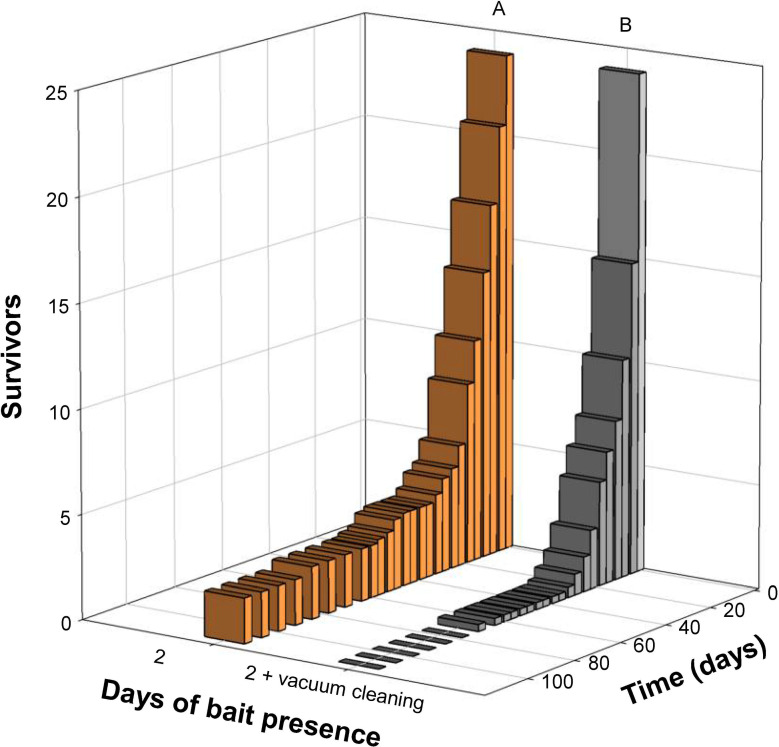
Survival of *Ctenolepisma longicaudatum* in population boxes of the 2-day treatments with alternative food sources and without alternative food sources due to vacuum cleaning. Different letters (A and B) indicate significant differences in survival between the treatments (Kaplan–Meier survival analyses, p < 0.05).

The treatment with bait present continuously for the first 8 days had a comparable survival to the discontinuous 8-day bait treatment (Kaplan–Meier; χ^2^ = 1.59, df = 1, p = 0.207; Fig 5). Removal of competing food sources increased mortality significantly in the 2-day treatments (Kaplan–Meier; χ^2^ = 27.60, df = 1, p < 0.001; Fig 6).

**Fig 7:**
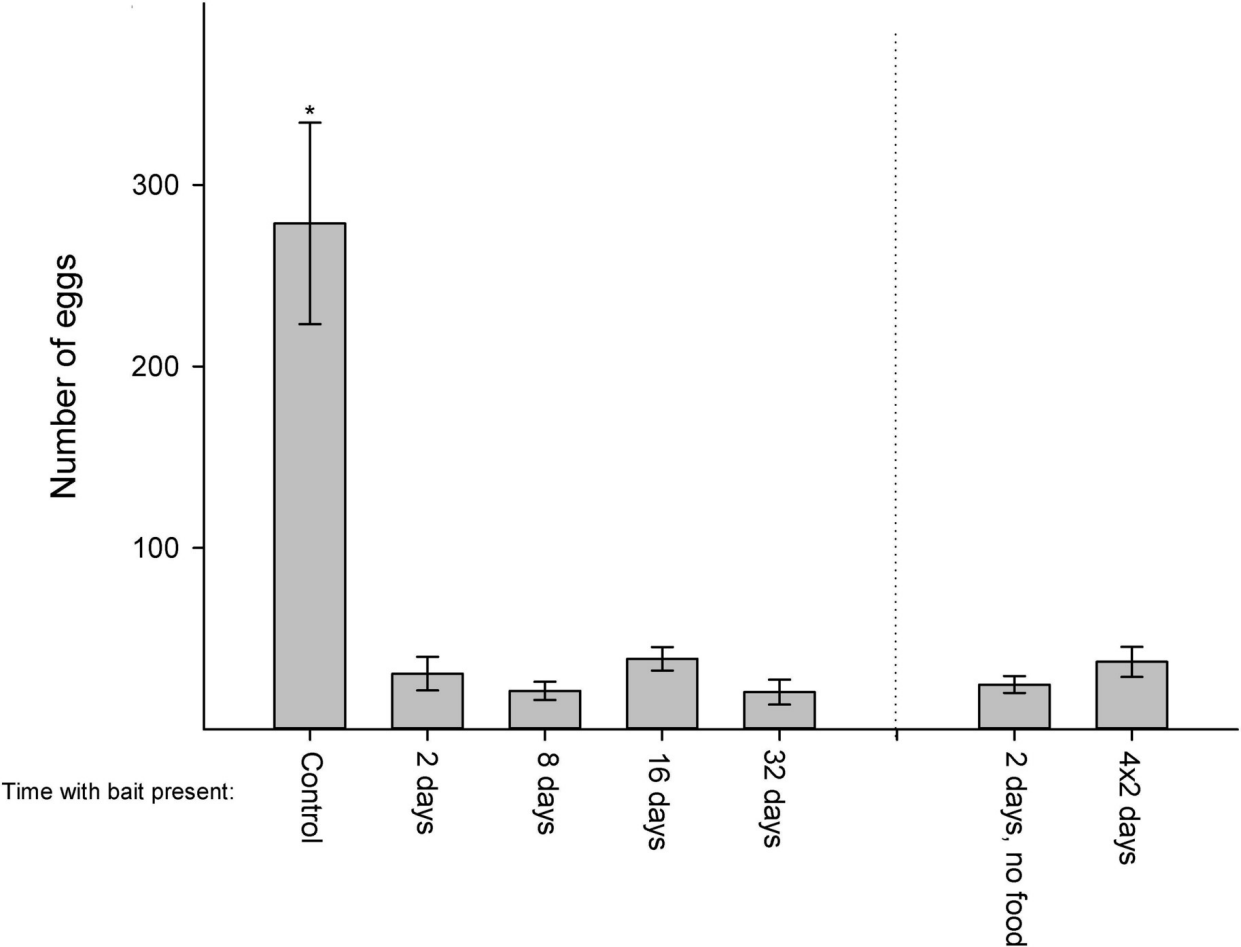
Egg deposition in treatments of different bait duration. * denotes significant difference to other treatments (Holm–Sidak, p < 0.05).

**Fig 8:**
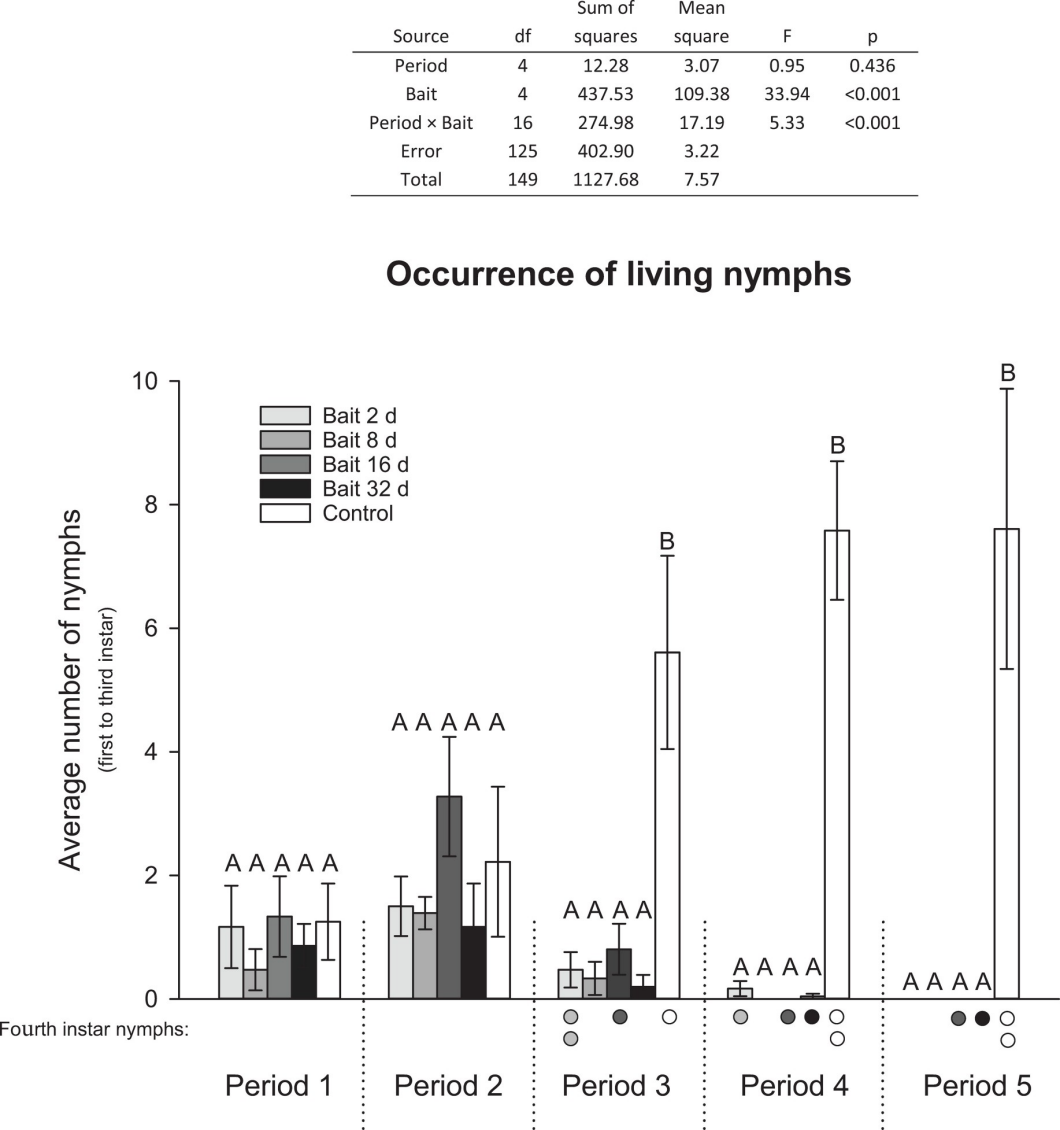
Average number of living *Ctenolepisma longicaudatum* nymphs (1^st^ to 3^rd^ instar) per population box (with 12 adults and 12 nymphs each) in different bait treatments at different periods. The table presents the two-way ANOVA that describes the effect of the two predictor variables: Period and Bait (number of days with bait present). Different letters (A and B) in the figure indicate significant differences between bait treatments within periods (Holm–Sidak, p < 0.05). Coloured dots below the x-axis indicate the number of 4^th^ instar nymphs that were present in the respective periods.

The numbers of eggs deposited in the bait treatments were significantly reduced by more than 85% compared to the control (ANOVA: *F* = 18.64, p < 0.001; Fig 7). Production of 1^st^ to 3^rd^ instar nymphs in the bait treatments was significantly affected in the last three periods (Fig 8). The bait-treated populations showed a decline in occurrence of nymphs, while there was an increasing number of nymphs in the control. In the last period, only the control had small nymphs in the initial three stages. In total, only six of the new juveniles managed to develop into the 4^th^ stage (first juvenile stage with scales), and they were observed in three out of the four treatments and in the control.

### Case studies–field observations on spatial elements

**Fig 9:**
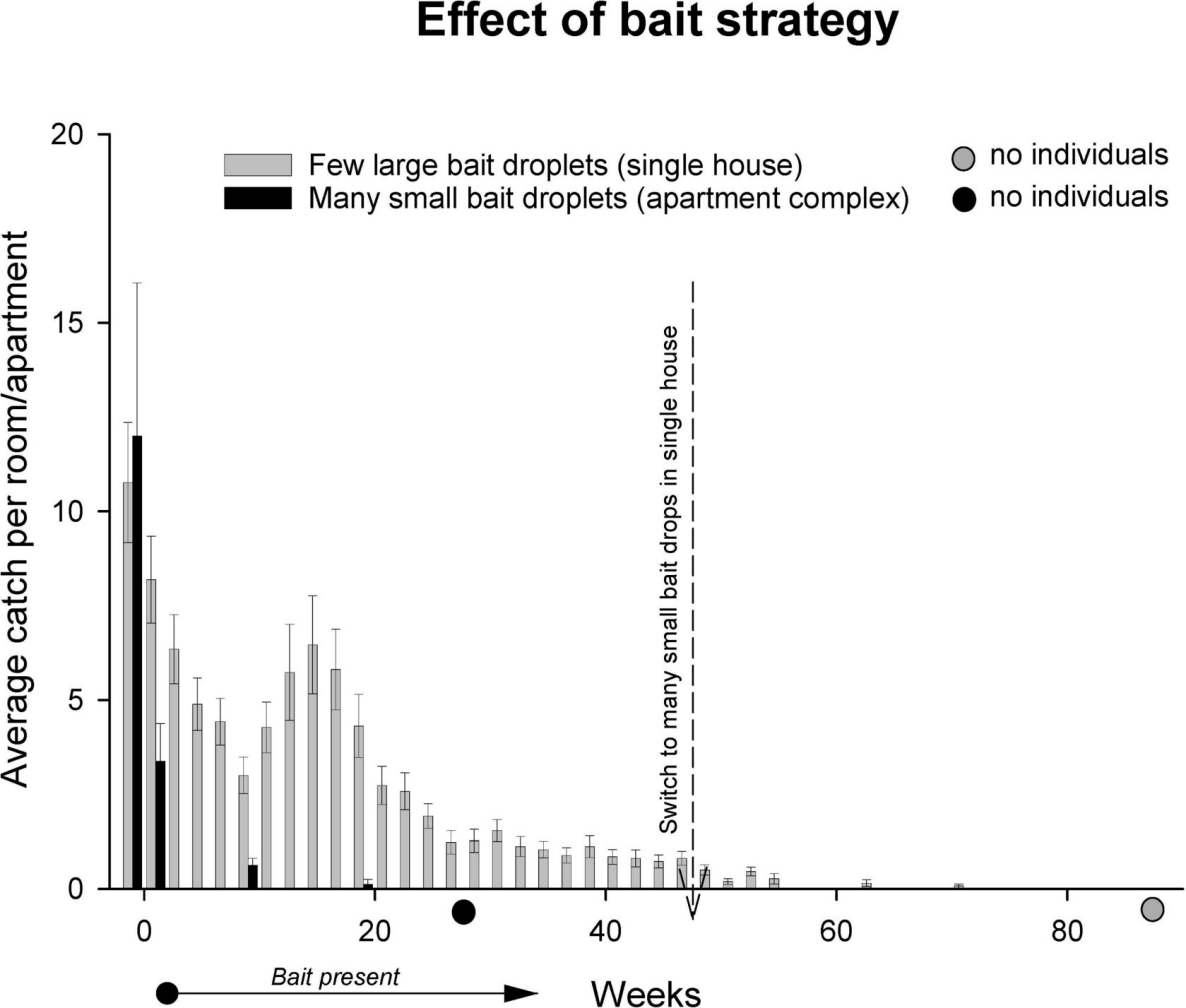
Population development of *Ctenolepisma longicaudatum* measured as individuals caught in sticky traps in a detached house and an apartment complex treated with poisoned bait. The single house was treated with bait stations until week 50, when many small droplets along wall skirtings were applied instead. The apartment complex received many small droplets along wall skirtings in the whole investigation period.

The detached house showed slowly progressing population decline when bait stations (few points of bait access) were present. After switching to widespread distribution of bait (many small droplets distributed along the wall skirtings) the population disappeared after an additional 25–40 weeks (Fig 9). The apartment complex, treated with widespread bait droplets for the whole treatment period, showed a faster decline compared to the single house and the population disappeared after less than 28 weeks.

## Discussion

In the present study, we showed that both spatial and temporal elements connected to the use of baits influenced the survival of *C*. *longicaudatum*. An extended availability of baits in both space and time, and the removal of alternative food sources increase control efficiency. Even though we lacked infested localities for direct comparisons to the observed field effects, the two case studies align with previously conducted field experiments [17] to underline bait as a solution. Field populations of *C*. *longicaudatum* are also anticipated to be severely affected by baits because all stages experienced high levels of mortality in the laboratory, and we observed strongly hampered egg deposition and nymph recruitment.

An important applied aspect detected in this study is connected to the behaviour of *C*. *longicaudatum*. The observed variable effects of different bait distributions pinpoint the importance of a sound spatial strategy during control efforts. The spatial use of the indoor habitat may differ between pest species, and cockroaches are good examples of applied bait considerations used for maximised effect [34]. They have a partly restricted distribution associated with repetitive use of feeding places, access to mating, safety or environmental stability in their aggregations, and consequently, they respond well to bait tactics adapted to that particular biological system [20,32,34]. Pharaoh ants, a second example, are even more extreme in this respect with their social organisation around food sources and are effectively controlled if ant trails are established between baits and the nests [45,46]. There is limited knowledge regarding foraging strategies in *C*. *longicaudatum*, but the improved efficacy by use of a wide distribution of bait along edges may point at a search for widely dispersed food sources. They may typically use fungi, plant materials, food leftovers and dead insects [18,19] found at several locations in a building, and they may even utilise paper as an energy source [47] if other more complete nutrition becomes scarce. During foraging, *C*. *longicaudatum* is likely to follow the edges that are difficult to traverse, and a food-reward is most likely encountered on the floor along edges where leftovers will accumulate. Moving along edges will also keep the silverfish in closer proximity to potential hiding places than if they roam more freely away from edges. Food search along edges will therefore lead to bait encounter during control if baits are systematically placed at these locations. Baits may also be placed in cracks and crevices to reduce exposure risk of pets and humans and prevent the bait from being removed during cleaning. Such an approach also seems reasonable because *C*. *longicaudatum* may have a wide distribution in apartments and is often found in most units and rooms in apartment buildings [17]. A strategy of treating fewer locations with large bait drops or bait stations, which works well with ants and cockroaches, may be futile for *C*. *longicaudatum* control as it may miss a large proportion of the population.

From a control point of view, the life cycle of *C*. *longicaudatum* appears to be well suited for decimation with baits. Thirteen instars must be passed before maturity, and at normal indoor temperatures, it takes at least 18 months before reproduction can commence [13,30]. All instars, except for the 1^st^, must also consume a meal before moulting to the next stage [30]. This enforces several occasions across different instars over a long period, during which bait can potentially be ingested, to increase the probability of poisoning. Such mechanisms are also expected to be at play in our laboratory study with increased mortality if bait presence exceeded 8 days. This effect is likely connected to increased encounter probability through ingestion of more than one meal. Manipulation of the indoor environment through removal of food sources supplying the pest population is also easy [19]. This is important for bait efficacy in cockroach control [32–35], and for *C*. *longicaudatum*, with its broad diet. Systematic vacuum cleaning prior to and during control combined with securing of food stock in closed containers are therefore likely to make a substantial difference. Even though life expectancy on filter paper alone is more than a year in *C*. *longicaudatum* [30], our laboratory experiment clearly showed increased efficacy when all competing food except paper was removed, and the “thoroughly cleaned” experimental treatment was actually the only test yielding complete eradication. This benefit is probably a result of increased bait consumption, but may also stem from a higher potency of pesticides in starved compared to recently fed insects [48–50].

Even though a large proportion of *C*. *longicaudatum* died within a few weeks, we observed a long period with a few survivors present in the population boxes. Such an extended period before total eradication has also been observed in studies of field management and is most likely connected to interactions among feeding habits, dispersal and habitat use [17], but may also stem from the moulting mechanisms in *C*. *longicaudatum*. Some specimens, in both laboratory and field populations, are likely to have entered their physiologically passive and non-eating state preceding moulting. This passivity may last for the final one-third of the current developmental stage [30] and will obviously delay the bait effect among these individuals. When combined with chance encounters of competing food sources instead of the bait, it explains the lack of 100% mortality in several of our experimental laboratory treatments. In field situations, this aspect of a delayed effect strengthens the argument for frequent, thorough vacuum cleaning to ensure adequate bait consumption among individuals in different development phases to evoke robust population declines, but this also advocates patience. Bait droplets have shown field functionality for at least 6 months after placement [27] and may consequently contribute towards total termination of the infestation in the long run. Short-term treatments with removal of deposited bait or bait stations can allow passive individuals to miss the opportunity to consume the bait and subsequently give rebounding infestations.

The laboratory investigations yielded significant differences with the duration of bait presence. From an applied point of view, these discrepancies may have a limited practical impact because bait will mostly stay in place in apartments until eaten by the pest insect. The difference in survival between short (2 or 8 days) and long (16 or 32 days) bait presence in the experiments does however highlight that removal of baits through cleaning should be avoided. It is also interesting that all experimental populations experienced a strong initial mortality followed by a long period of slow and gradual decline. Contrary to the no-bait treatment, we observed mortality among the initial individual’s months after removal of the bait from the laboratory cultures. This indicates a contribution from secondary poisoning as the dead individuals were left in the population boxes and consumed by the remaining conspecifics. The laboratory declines also mirror field observations during *C*. *longicaudatum* management [17], and it is reasonable to assume that dead individuals may play an important part in the distribution of the insecticide through secondary poisoning [27] also under field conditions. Modern buildings typically contain an assembly of cavities, ducts and tubes for electrical systems, built-in installations and ventilation systems, and it is possible that the observations of building-wide distribution are supported by this multidirectional and partially hidden dispersal grid [15,17]. In this respect, the delayed mortality from indoxacarb may be beneficial as it kills the insects through a delayed bioactivation of the toxins several hours after consumption of the bait [51–53]. Bait eating and subsequent retreat to hideouts may consequently bring toxins into the hidden locations and the pathways used for dispersal to result in a preventive effect. Returning populations due to failed treatments or new introductions have thus far not been observed in field management situations monitored for as much as a year [17], but studies with an even longer time frame are needed to confirm any preventive benefits.

The bait used in this study is intended for cockroach control but also appears efficient against *C*. *longicaudatum*. The nutritional ingredients in the bait are a trade secret, but it is reasonable to assume that it contains sugars/carbohydrates and other important dietary elements. It is likely that baits developed specifically for Lepismatid species can improve the effect from baits. Dietary preferences change from carbohydrates to proteins at increasing temperatures in *L*. *saccharinum* [54], and the functionality of a bait might therefore vary with temperature, and perhaps other factors like the species in question, developmental stage or gender. Such potential specificity and individual disparity in expected consumption and subsequent control effect is a challenge that should be addressed by future comparative studies among the encountered indoor bristletail species. Fortunately, most bristletail species appear to prefer sugars, thus assuring some consumption of sacchariferous baits. Finally, an incorporation of olfactory food signals [55,56] into the bait matrix may be utilised to make a silverfish control strategy even more effective with less effort.

## Conclusions

The present study supports poisoned bait as an efficient and safe approach to control *C*. *longicaudatum* when combined with sanitation. A long duration of bait presence at the infested locality in combination with a wide distribution of small bait droplets seem to be crucial to handle the slow and cryptic lifecycle of *C*. *longicaudatum*, or to reach stragglers hidden in complex constructional parts of a building. Improved baits and application strategies may advance efficacy further, but this study indicate beneficial, safe and cost-efficient effects from the use of commercially available baits.
